# Sand spikes pinpoint powerful palaeoseismicity

**DOI:** 10.1038/s41467-021-27061-6

**Published:** 2021-11-18

**Authors:** Elmar Buchner, Volker J. Sach, Martin Schmieder

**Affiliations:** 1 HNU - Neu-Ulm University of Applied Sciences, Wileystrasse 1, D-89231 Neu-Ulm, Germany; 2 Meteorkrater-Museum Steinheim, D-89555 Steinheim am Albuch, Germany; 3 Fokus Natur, Am Heselsberg 29, D-88416 Ochsenhausen, Germany

**Keywords:** Natural hazards, Sedimentology

## Abstract

Sand spikes, pin-shaped, carbonate-cemented sandstone bodies of variable size widely interpreted as sedimentary concretions, have been enigmatic for nearly two centuries. We here present a high-energy mechanism for their formation. Two classic sand spike occurrences are found in the North Alpine Foreland Basin of Central Europe and at Mount Signal in southern California, USA. A distinct seismite horizon in Mid-Miocene Molasse sediments of southern Germany, genetically linked with the Ries impact event, exhibits dewatering structures and contains numerous sand spikes with tails systematically orientated away from the Ries crater. Sand spikes at Mount Signal, strikingly similar in shape to those found in Germany, have tails that point away from the nearby San Andreas Fault. Based on their structural and stratigraphic context, we interpret sand spikes as a new type of seismite and a promising tool to identify strong impact-induced or tectonic palaeo-earthquakes and their source regions in the geologic record.

## Abstract (web summary)

Sand spikes, sandstone bodies that have been enigmatic for nearly two centuries, represent a new type of seismite and a promising tool to identify strong impact-induced or tectonic paleo-earthquakes and their source regions in the geologic record.

## Introduction

Sand spikes are eye-catching sedimentary features that have been enigmatic for about two centuries. According to the description by Nichols^1^ in 1906, they “take the form of an irregularly botryoidal ball from which projects a stout, tapering stem in such wise that the object assumes the shape and proportions of an ancient mace.” However, as Sanborn^2^ put it 70 years later, “No discussion of concretions would be complete if mention were not made of a real puzzler, a truly unique type of concretion that apparently remains unexplained—sand spikes.” Over the decades, a variety of theories have attempted to explain the mechanism behind the formation of sand spikes, for which two classic occurrences are known: (i) the North Alpine Foreland Basin (NAFB) of southern Germany, from which the so-called “Zapfensande” were reported in Molasse sediments as early as in the 1820s^3–6^, and (ii) Mount Signal in the Imperial Valley of southern California, USA, where sand spikes were discovered mainly in the 1930s to 50s^1,7–9^. The NAFB and Mount Signal sand spikes are strikingly similar in their appearance, structure, composition, and internal texture^1–9^. Previous theories interpreted sand spikes as possible stalactites^1,4,7^, petrified seaweed, turnip or mushroom^7^, fulgurites^2^, sand-filled crab burrows (i.e., trace fossils)^9^, and elongated concretions that formed either within longshore currents below the tide level^8^ or in alignment with the subsurface groundwater flow^6^.

In the present study, we propose a high-energy, event-related formation mechanism for sand spikes in Germany and California (as well as potential sand spikes elsewhere, see Supplementary Table 1). As we will show, sand spikes seem to occur preferably in geologic settings that offer the potential of major seismic shaking: while the sand spikes found at multiple locations across the NAFB appear to be genetically linked to the 24 km-diameter Nördlinger Ries impact crater, sand spikes from California occur in sediments only a few tens of km west of the San Andreas fault system, one of the most tectonically active and earthquake-prone geologic regions on our planet.

Large impact events impart a significant portion of their energy into the target rock, thereby causing intense, shallow earthquakes with a great potential for environmental destruction^10–16^. Impact-triggered earthquakes produce seismites in extensive volumes of surface-near sediment that are in many ways similar to seismites generated by tectonic earthquakes^14–17^. The style of deformation is mainly governed by the nature of the near-surface substrate, i.e., grain size, water saturation, diagenesis/cementation, and other factors influence the formation and final character of the seismite^15,18^. Field evidence for major impact-triggered earthquakes has been gathered from the wider surroundings of a number of terrestrial impact sites^11,13,15,16,19^.

The present study elaborates the regional-scale seismic effects of the Ries impact, which has a well-constrained Mid-Miocene (Langhian) ^40^Ar/^39^Ar age of 14.808 ± 0.038 Ma^20,21^, making it a few kyr older than the nearby Steinheim impact (Serravallian)^16,22^. In addition to proximal impact ejecta and tektites (moldavites)^23^, a coarse-grained layer of distal Ries ejecta (predominantly pebbles, cobbles, and boulders of Upper Jurassic limestone) forms a distinct stratigraphic marker bed within Neogene Molasse sediments of the NAFB^16,24–29^. The ejecta layer, with clasts ballistically transported over at least 180 km from the Ries crater and preserved in fine-grained siliciclastics^16,24,26,29^, is at several localities within the NAFB associated with an underlying seismite unit produced by the Ries impact-earthquake^16^. The seismite, characterized by distinct soft-sediment deformation, provides evidence for the high energy released during impact-triggered seismic shaking within a radial distance of >200 km from the impact site^15,16,19,28^.

As we will demonstrate, sand spikes, which are known form the NAFB of southern Germany and Mount Signal in southern California, represent a new type of seismite produced in volumes of water-saturated, unconsolidated sand during major earthquakes triggered by asteroid impact events (such as the Ries impact) or strong tectonic activity (e.g., along the San Andreas Fault). Where preserved in situ, sand spikes help constrain the location of (paleo-)seismic sources and may be a useful tool to assess the seismic hazard potential of tectonically active regions on Earth.

## Results and discussion

### Sand spikes in southern Germany

**Fig. 1:**
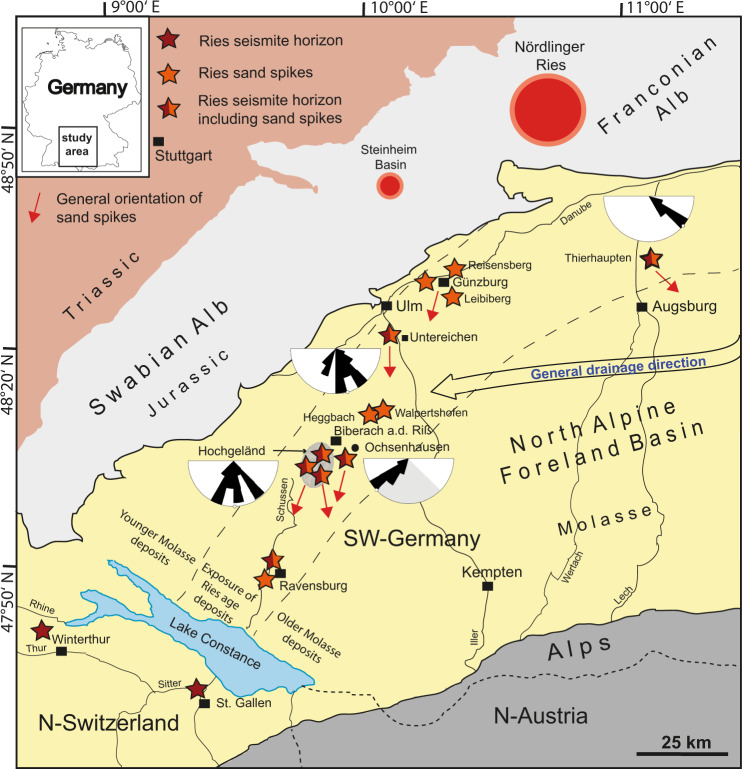
Geographic and geologic situation in the study area in southern Germany and adjacent countries. Outcrops with seismites and/or sand spikes linked to the Ries impact are situated within a distance of 40 to 205 km from the center of the impact structure. The orientation of sand spike tail apices (orange arrows and small rose diagrams) is indicated where measured. It is noteworthy that the orientation does not align with the general drainage direction in the Foreland Basin at the time of the Upper Freshwater Molasse.

Sand spikes (locally referred to as “Zapfen” within the Zapfensande)^4–6,25,30–32^ are unique sedimentary features in Molasse sediments of the NAFB. They are known from various historic and active sand pits, as well as natural outcrops in the western and central part of the NAFB. Sand spikes are found in the area around Ravensburg^15,16,24^, in ravines and gullies in the Hochgeländ plateau^15,16^, at Ochsenhausen near Biberach an der Riss (Biberach a. d. Riss)^6,16^, between Biberach and Ulm^25,31–33^, as well as near Günzburg^32^ and Thierhaupten^31^. They occur as single, arrow-shaped specimens and as spike- or board-shaped aggregates. Individual sand spikes typically have a bulbous head, from which an outward-thinning tail (stem) branches off. The size of individual sand spikes ranges from a few to several tens of centimeters and, rarely, more than a meter. The majority of spike tails in the NAFB are orientated more or less southward^6^, with local variations to the southwest (SW) and southeast (SE). Most notably, all sand spike tails seem to point away from the Ries crater (Fig. 1).

**Fig. 2:**
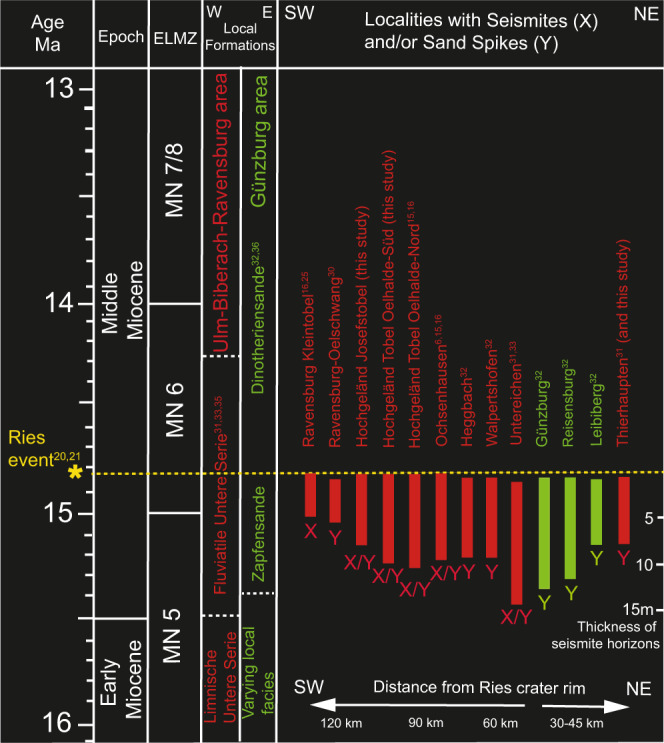
Stratigraphic constraints on the age and correlation of sand spikes (Zapfensande) in southern Germany. Indicated are known localities with significant occurrences of sand spikes in the Upper Freshwater Molasse of the western and the central North Alpine Foreland Basin region, southern Germany, and the stratigraphic position of the sand spike-bearing deposits (Local Formation names) with respect to the European Land Mammal Zones (ELMZ) and the Ries impact event. All sand spikes occur in surface-near, unconsolidated pre-Ries deposits.

The Molasse sediments of the NAFB, clastic deposits of Oligocene to Miocene ages, are subdivided into two marine-terrestrial megasequences^33^, the Lower Marine and the Lower Freshwater Molasse, and the Upper Marine and the Upper Freshwater Molasse (henceforth UFM)^31,34,35^. Each of these sequences contains thick sandy units that are typically unsolidified or weakly consolidated. Stratigraphically, the sand spike-bearing deposits belong to the “Fluviatile Untere Serie” (or the Zapfensande) within the Middle Miocene UFM^31,33^ (Fig. 2). The Fluviatile Untere Serie reflects fluvio-lacustrine conditions during the final cycle of clastic sedimentation within the NAFB^33^ (Fig. 1). According to the older literature, sand spikes appeared to spread across a wide stratigraphic range within Middle to Upper Miocene deposits^32^. Consequently, their formation seemed more or less random and did not appear to be linked to any particular time interval or an instantaneous event. However, this view has recently changed. In the more recent literature, the Zapfensande have consistently been assigned an early Middle Miocene (Langhian) (bio-)stratigraphic age (corresponding to the Eppelsheim Formation)^33,36^. Thus, they represent an eye-catching sandstone marker horizon within up to ~5000 m of Molasse sediments in the NAFB^34,35^.

Distinct soft-sediment deformation features were recently described from the former construction site at Liebherr Ochsenhausen, in the Hochgeländ area, and near Ravensburg^15,16,25^. The structural inventory of soft-sediment deformation includes meter-sized slumps, convolute bedding, ball-and-pillow and flame structures, and clastic dikes. These features are consistent with their formation in a seismite produced during a large paleoearthquake^11,14,16,37–39^. The dip of the slumps and the strike of slump axes are consistent with a seismic source located in the northeast or north-northeast, i.e., the Ries crater region. The genetic link between the seismite and the Ries impact is evidenced by a marker horizon of coarse-grained distal Ries ejecta including shatter-coned limestone fragments and shocked quartz grains^16,24–27^ that caps the seismite. Outcrop-scale soft-sediment deformation, including fossil sand diapirs as far south as northern Switzerland, occurred within a radial distance >200 km from the Ries crater^16,19,28^.

**Fig. 3:**
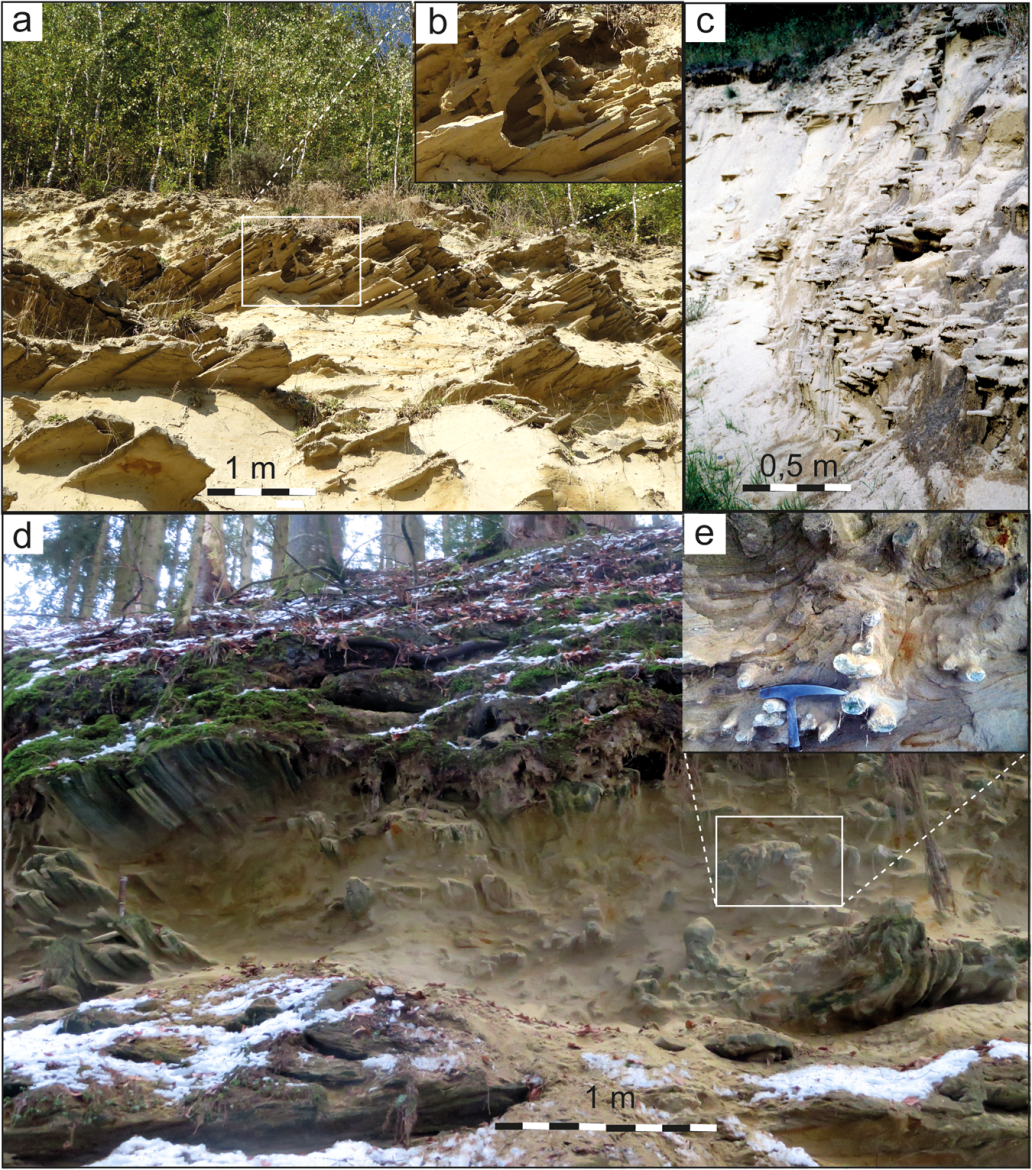
Outcrops with seismites and/or sand spikes in the Upper Freshwater Molasse. **a** Sand- and claypit Untereichen south of Ulm. Sand spikes often occur as board-like aggregates parallel to layering of the sandy deposits or as single arrow-like specimens (**b**). **c** Former outcrop “Liebherr” in Ochsenhausen near Biberach an der Riss exhibiting board-like aggregates of sand spikes parallel to the layering of the sandy deposits or as single arrow-like specimens (lower right). The outcrop with sand spikes interfingers laterally with sandy deposits showing convolute bedding and slump structures. **d** Outcrop “Tobel Oelhalde-Süd” in the Hochgeländ area south of Biberach an der Riss. Board-like aggregates of sand spikes (left) and single specimens (**e**) occur within sand deposits showing convolute bedding, sand diapirs, and sand blows. **e** Sand spikes tracing convolute bedding faults. All photographs taken by V.J.S.

**Fig. 4:**
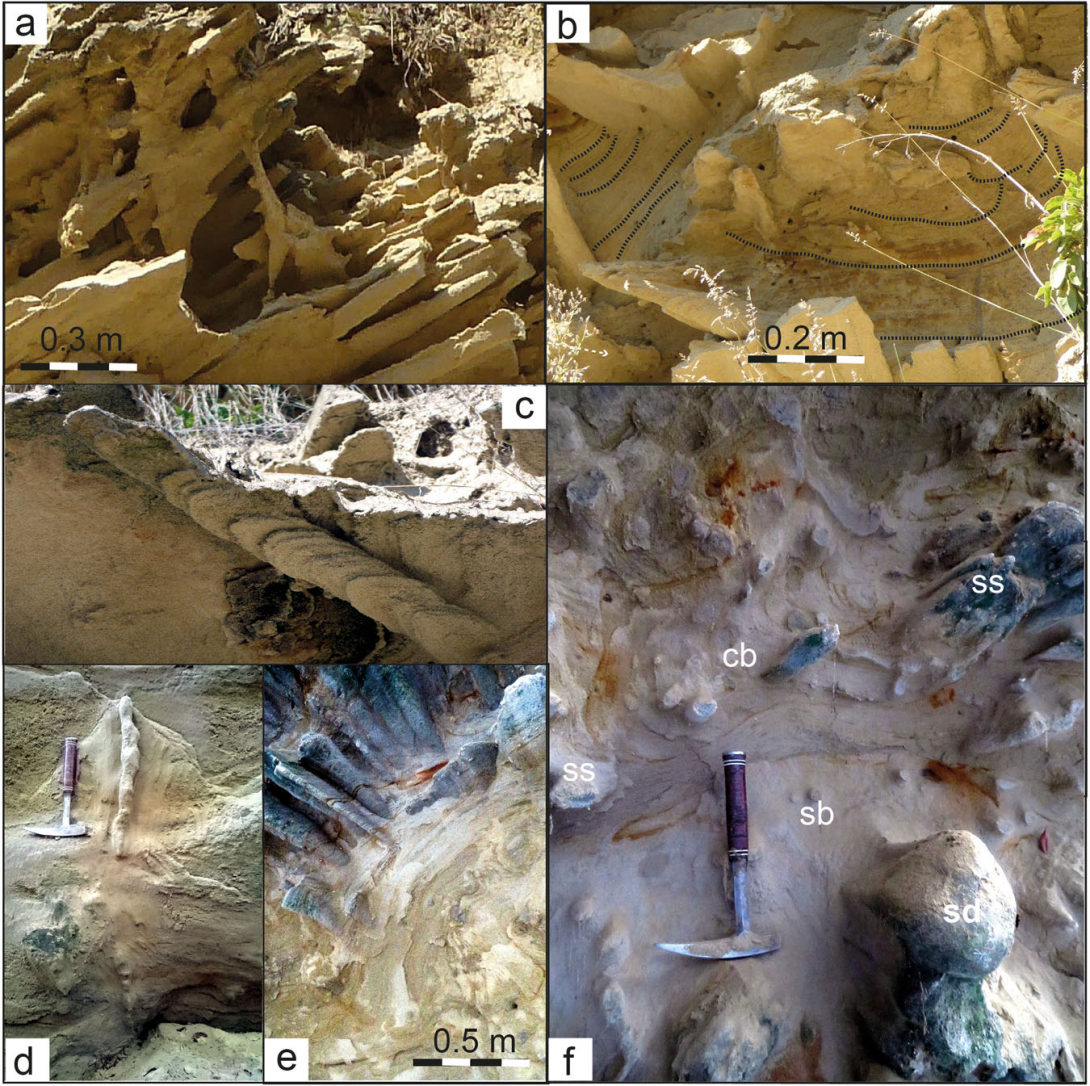
Ries seismite and sand spikes in the Upper Freshwater Molasse near Ulm and Biberach a. d. Riss. **a**–**c** Sand spikes in the Untereichen sand- and claypit south of Ulm. **a** Board-like and single arrow-like sand spikes and a vertical dike-like sand spike (near center of the image). **b** Sand spikes in seismite deposits with convolute bedding. **c** A horizontally arranged sand spike perpendicular to two vertical board-like sand spikes in the background. **d** Dike-like sand spike in loose sands at the “Tobel Oelhalde-Nord” in the Hochgeländ area south of Biberach. **e**, **f** Sand spikes associated with seismites in the outcrop “Tobel Oelhalde-Süd” in the Hochgeländ area. **e** Sand spikes orientated along a slump fault. **f** Sand spikes (ss) in a seismite horizon showing convolute bedding (cb), sand blows (sb), and a sand diapir (sd). All photographs by V.J.S.

**Fig. 5:**
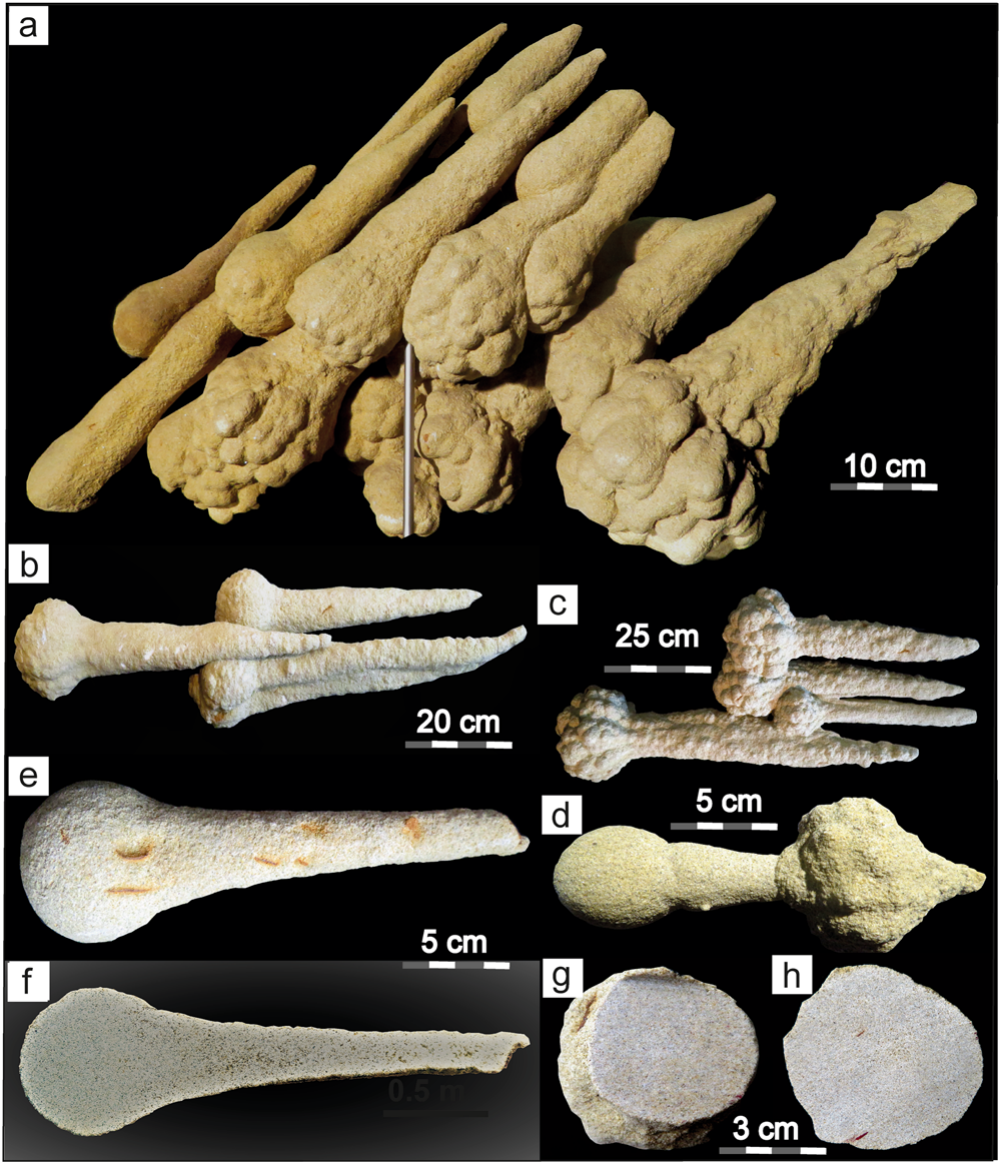
Museum-quality sand spikes from the former Liebherr outcrop in Ochsenhausen. **a** Typical sand spike aggregate with roundish to cauliflower-like heads and uniformly orientated tails. **b**, **c** Sand spike aggregates with roundish (**b**) and cauliflower-textured (**c**) heads and uniformly orientated tails. The tail of the left individual in **b** is slightly deformed by the sand spike to the right. **d** Sand spike individuals seemingly formed during a protracted, dynamic process. The sand spike to the left speared and deformed the sand spike to the right. **e**, **f** A typical nail-like sand spike showing the surface (**e**) of the cut specimen and the interior side of the same sand spike cut in half. It is noteworthy that the sand spike head is more densely cemented than the spike tail that contains some visible open pore space. **g**, **h** Two heads of sand spikes cut in two. The interior of the sand spikes does not show any structural features or concretionary cores. All photographs taken by V.J.S. Sand spikes in **a** and **d** are shown with approval by the Braith-Mali-Museum in Biberach a. d. Riss where these specimens are on display.

We describe, in particular, sand spike-bearing deposits in the sandpit Untereichen (Figs. 3a, b and 4a–c) south of Ulm^31,33^, in the ravines “Tobel Oelhalde-Nord”^15,16^ (Fig. 4d) and “Tobel Oelhalde-Süd” (this study, see Supplementary Material and Figs. 3d, e and 4e, f) south of Biberach a. d. Riss, and in the historic Liebherr outcrops in Ochsenhausen^6,15,16,25^ (Figs. 3c and 5a–h). We chose these localities to test whether the Zapfensand deposits are, in fact, part of the extensive Ries seismite recently recognized in the NAFB^16^.

In the Untereichen sandpit, sand spikes occur within the Early to Middle Miocene Fluviatile Untere Serie^31,33^. Biostratigraphically, these deposits are part of the European Land Mammal Zone (MN) 5^33,40^. The Ries impact event (~14.81 Ma^20,21^) occurred around the transition from MN 5 to MN 6^15,25,33,36^; hence, strata exposed at Untereichen biostratigraphically (slightly) predate the Ries impact^31^. Distal Ries ejecta would be expected a few meters higher up in the section, but are not preserved in this outcrop^33^. However, in the ~15 m-thick sandy deposits of the Fluviatile Untere Serie^31^, several layered and board-like aggregates, and numerous individual sand spikes can be observed. Most parts of the unconsolidated sands exibit intact fluvial sediment structures, but slump structures and convolute bedding coupled with sand spikes also occur (Figs. 3a, b and 4a–c, and Supplementary File S1).

A macro- to megascopic seismite horizon in the Hochgeländ plateau south of Biberach has also been genetically linked to a strong paleoearthquake induced by the Ries impact^15,16^. Some exposures of the seismite exhibit conspicuous dewatering structures^16^; however, sand spikes are only found locally. In other sections of the Ries seismite, such as the “Tobel Oelhalde-Nord” and, in particular, the “Tobel Oelhalde-Süd” (Supplementary Material), board- and arrow-shaped sand spikes occur (Figs. 3d, e and 4e, f). The sand spikes are preferably concentrated within slump folds and in outcrop sections characterized by convolute bedding. In these domains, sand spikes predominantly occur within fold axes, i.e., a compressive lithoregime. The largely unconsolidated seismite in the Hochgeländ area attains up to ~10 m in thickness. It is overlain by distal Ries ejecta and, therefore, must have formed (shortly) before the Ries impact^24,25^.

The former outcrop “Liebherr” in Ochsenhausen^6,25^ also featured a seismite horizon with distinct dewatering structures^16^ that laterally interfingers with portions of the host sand unit that are seemingly undisturbed. In addition, meter-scale slump structures occur in the same stratigraphic horizon (Supplementary Fig. 2). A large number of sand spikes quite variable in size and shape were discovered in the ~10 m-thick sands between the 1960s^6^ and 1990s, during construction and expansion of the Liebherr factory. Board- and arrow-like sand spikes and slumps occur in the same stratigraphic level. Many of the specimens were documented in situ and are today stored in private collections and on display in a local museum in Biberach (Fig. 5). Field investigations by one of the authors (V.J.S.) suggest the sand spike-bearing seismite is overlain by a reworked horizon of distal Ries ejecta. Therefore, the seismite exposed at Ochsenhausen^6^, again, must have formed from surface-near, unconsolidated sediments that predate the Ries impact.

### Structure, composition, and orientation of sand spikes

**Fig. 6:**
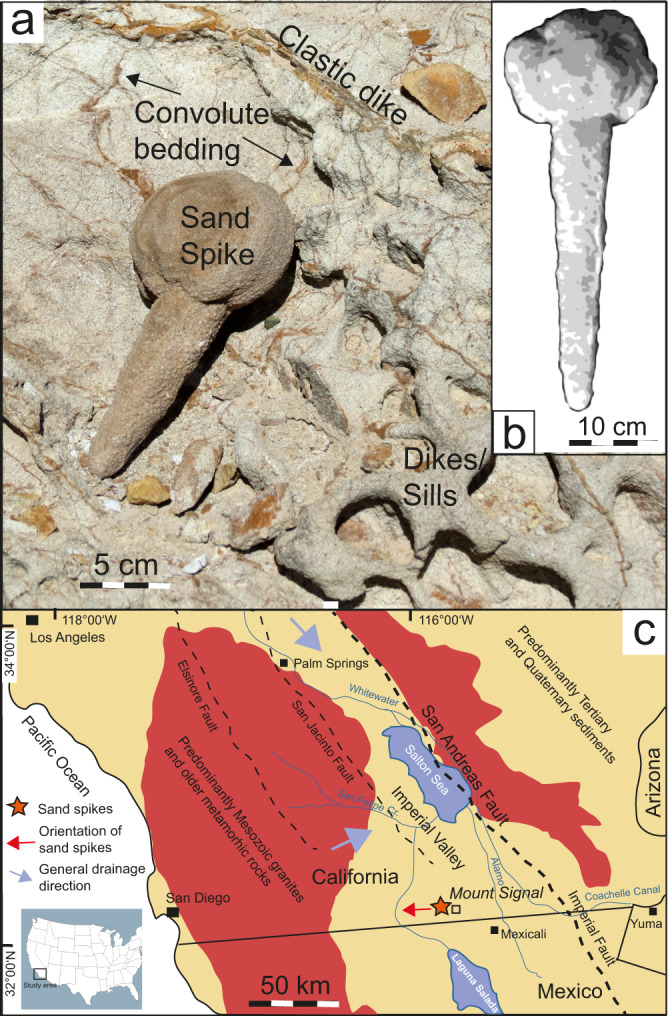
The sand spike occurrence near Mount Signal in southern California, USA. **a** Typical sand spike from Mount Signal, California. The sand spikes occurs within a horizon with convolute bedding, dikes, and sills (image: Mila Zinkova, USRA Earth Science Picture of the Day, 24 November 2013; https://epod.usra.edu/blog/2013/11/sand-spike-concretions.html). **b** Sand spike from Mount Signal described by Garner (1936; redrawn after his Fig. 4). **c** Simplified geological situation in the Imperial Valley, California, with the sand spike occurrence at Mount Signal and the most prominent faults of the San Andreas transform fault system indicated. Apices of the sand spikes are orientated away from the San Andreas fault system and perpendicular (or even opposite) to the general drainage system in the Imperial Valley.

Sand spikes commonly appear as single individuals with a bulbous head and an outward-thinning, tail-like protrusion (Figs. 4c, 5e, f, and 6), but can also occur as board- or sill-like aggregates parallel to the general layering of their host sands (Figs. 3c, 4a, and 5a). They often occur in stacked layers, e.g., at Untereichen^31,33^ (Fig. 3a, b) and the former Liebherr site in Ochsenhausen^6,25^ (Figs. 2 and 3c). The spike head can be almost perfectly round or can have an outer cauliflower-like texture^6^. The surface of the spike’s tail can be smooth or pitted. Rarely, “sand spike couples” are observed where a first-generation sand spike seems to dynamically interact with a second-generation sand spike (Fig. 5d). The similarity between most sand spikes suggests they form by a non-random mechanism that generates their structural and textural features in a certain systematic, geologically and physically controlled, manner.

According to an analysis of specimens from Ochsenhausen, the sand spikes consist of quartz (70–80%), feldspar (10–12%), mica (6–9%), and accessory minerals (e.g., garnet, epidote, and apatite) in the fine- to medium-grained sand fraction and are essentially identical in modal composition and grain size with their host deposits; the latter locally contain rare fossils, such as mammal bones; lignite; secondary minerals and cements, e.g., sandy sparry calcite; and are in places ferruginous and/or display Liesegang bands^6^. The majority of sand spikes are cemented by calcite [Ca/(Fe + Mg) = 3.2, with minor additional Na and K], which typically makes up ~30–40% of the sand spike volume. Sulfates and other cements are negligible^6^. Although widely interpreted as sand concretions^5,6,31–33^, the NAFB sand spikes exhibit no concentric growth structures around an inner core (e.g., fossils or rock fragments), which are considered hallmarks of concretions^41–43^. They are internally featureless and cement-supported^6^ (Fig. 5e–h). A notable internal feature of the sand spikes is that their heads typically appear more compact than the adjacent tails that commonly contain some open pore space (Fig. 5e, f). Some sand spike aggregates exhibit complex external deformational features. As depicted in Fig. 5b, the head of one sand spike appears to slightly bend the tail of another individual. In Fig. 5d, the sand spike shown on the left seems to spear, deform, and distend its counterpart on the right. These observations (also noted in sand spikes from California^8^) suggest that the interacting sand spikes formed in at least two episodes and that the deformed first-generation sand spike must have been soft and plastic when it was overprinted by the second-generation sand spike.

We measured the orientation of 311 individual sand spikes (Fig. 1 and Supplementary Fig. 5), including sparse orientation data from the literature^6^. The great majority of sand spike tails point in the same general direction, with a natural variance within ≤90°. At Ochsenhausen, they are generally orientated in a southward (S to SW) direction (mean azimuth 226°, *n* = 50 sand spikes); additional spike tails that are no longer exposed apparently had a SE- to S-pointing direction^6^ (Fig. 1). At Untereichen (mean azimuth 177°, *n* = 93) and in the Hochgeländ (mean azimuth 165°, *n* = 128), the spike tail azimuth is also chiefly towards the S (Fig. 1), with a natural variance from SW to SE. In the Thierhaupten sandpit, spike tails point SE (mean azimuth 131°, *n* = 40). We keep in mind that the direction of fluvial drainage and sediment transport within the UFM in this region was generally orientated E-W during the Mid-Miocene^31,34,35,44^. In other words, the regional surface and groundwater paleo-flow vectors are more or less perpendicular to and, therefore, remarkably inconsistent with the alignment of sand spikes in the NAFB (Fig. 1).

### Sand spikes from Mount Signal, California

Sand spikes similar in shape and size to those from the NAFB were discovered in the 1930s in late Neogene to early Quatenary sands and gravels at Mount Signal (Fig. 6), close to the Mexican border in the Imperial Valley of South California^1,2,7–9^. In the following years, thousands of sand spike specimens were found at that particular site, but nearly all of them were collected or inadvertently crushed, mostly by bulldozers in the 1950s^2^. Today, the sand spike occurrence seems to be exhausted.

The Californian sand spikes, whose origin is also still debated as outlined in the introductory note^1,2^, range in size from about 3 to 33 cm and occur in a variety of shapes^2^. Some spikes come with heads that are perfectly round, while others are rather knobby to cauliflower-textured (Fig. 6)^1,2,7–9^, strikingly similar to sand spikes from the NAFB (Fig. 5)^6^. The Mount Signal sand spikes are composed of the sand that also forms the hillocks in which they occur. Sand spike heads consist of solid sandstone composed of quartz (~53%), feldspar (~2.5%), biotite, and iron oxide (~6%)^8^, and contain no fossil or other cores at their center nor do they feature any obvious internal sedimentary structures. Calcite cement constitutes ~30–50% of the sand spike volume^1,8^. Similar to the sand spikes from the NAFB, those from Mount Signal show a remarkably uniform orientation of their spike tails. Over 95% of the spike tails point west^2^.

### Sand spikes are not typical concretions

Although sand spikes are somewhat variable in size and shape across different localities, they generally show a very similar anatomy. A genetic blueprint seems evident for all sand spikes^2,6,8^, regardless whether they occur as single specimens or as groups or larger aggregates of interconnected spikes^6^. Their eye-catching shape—a bulbous head with an outward-thinning tail—appears to be unique to sand spikes and is not known from other types of concretions. Concretions are common geologic phenomena in almost all types of sediments and sedimentary rock, including sandstones, shales, siltstones, and limestones^41–43^. They form via mineral precipitation around a central nucleus, commonly (well-preserved) fossils, or other types of core material at their center, and typically exhibit a range of internal structures, such as spherical growth layers or septarian-like features. The most common type of concretions are spherical carbonate concretions. Concretion formation has been explained by diffusion and rapid syn-depositional reactions with organic solutes and other pore water constituents^41–43^. Compared to carbonate concretions, sand concretions are comparatively rare, particularly in continental sands. Iron oxide-cemented sand in the form of spheroids and pipe-like concretions, for example, were reported from the Navajo Sandstone in Utah, USA. These features exhibit, internally and externally, boxwork-like structures^45^. Giant spheroidal, calcite-cemented sand concretions (‘cannonballs’) were described, e.g., from the fluvial Mid-Cretaceous Dakota Sandstone in Kansas^46^ and in Cretaceous marine sandstones of Wyoming and Utah, USA^42^. Again, all of those sand concretions exhibit more or less distinct internal structures, including concentric rings/layers, (radial) septarian features, cone-in-cone structures, and other growth-related patterns^42,46^. The association of organic matter and microbial structures, as well as microbial by-products, suggests biogenic processes are the dominant mechanism for the (rapid) growth of calcite cement within sand concretions^41–43,45,46^.

A number of concretion-like sandstone bodies are reported in the literature that are internally structureless and bear a strong resemblance to the sand spikes described herein. Occurrences of such sandstone bodies are known from France^47,48^, Italy^49,50^, the United States^45,51,52^, and Australia^53^ (all listed in Supplementary Table 1) and may be worth (re-)investigating further with respect to their formation. Currently, both the appearance and formation of those sand spike-like features are explained in a more or less similar way. Elongated, rod-shaped and calcite-cemented sandstone bodies occur as isolated specimens and aggregates of interconnected individuals^53^. The spike-like sandstone bodies taper toward a preferential direction and often have enlarged or bulbous to cauliflower-like heads on the opposite side, which was in some studies explained as an indication of down-gradient growth^42,53^.

Two main models have been proposed to explain the formation of elongated sand concretions. Some authors favor an event-based model with increased flow velocities and gradients caused by a tectonic event^5,53^. However, a continuous flow model under steady groundwater flow conditions seems to be the more prevalent explanation^42^. Both models posit that sand concretions form within the phreatic zone (i.e., the sediment volume permanently submerged under the groundwater table), with their elongation parallel to the groundwater flow^45,52,53^. The geometry of concretions has been variably linked to paleo-groundwater flow vectors, in turn influenced by, e.g., tectonics, sea-level change, climatic oscillations, and shoreline erosion^53^. An additional possible mechanism was suggested to be the formation of sand spikes as trace fossils^9^. Although sand-filled animal (e.g., arthropod) burrows and tunnels are common in shallow-marine and tidal-coastal settings^54,55^, the subhorizontal arrangement of sand spikes in continental, surface-near fluvial sediments, their featureless internal texture, wide range of sizes (a few centimeters to over a meter in spike length), and systematic orientation argue against a biogenic origin.

Apart from sand spikes and sand-spike-like features, sand concretions without internal structures seem to be relatively rare. As pointed out above, sand spikes from the NAFB and Mount Signal are internally remarkably structureless and do not exhibit any type of core^2,6^ (Fig. 5e–h). We, therefore, suggest sand spikes are not typical sand concretions, as widely proposed previously. Rather, their outer appearance and inner texture is consistent with the sedimentary characteristics of sand injectites, a type of seismite that forms during earthquake-induced liquefaction at magnitudes *M*_W_ 5.5 and higher^37–39^. Fine-grained and internally featureless sand- and siltstone bodies of irregular shape, similar in appearance to sand spikes in the NAFB and their associated dikes and sills, are found, e.g., in the Paraná Basin of Brazil, where widespread seismites were linked to the large Permotriassic Araguainha impact^11^. Although the latter commonly exhibit gut-like structures and complex clastic dikes on an outcrop scale^11^, sand spikes may be a special type of sand-derived seismite. They likely represent dewatering and subhorizontal injection structures that formed from water-saturated, granular, and porous sediment under increased pore water pressure^17^. Interestingly, a somewhat similar tectono-seismic model for the formation of sand spikes was postulated by Rühl^5^ more than 120 years ago: he suggested tectonic activity in response to the Alpine orogeny may have been the trigger for the formation of the Zapfensande in the NAFB through enhanced fluvial erosion, flooding, and the wholesale deposition of sands in a ‘catastrophic’ event. Similarly, sand spike-like features in Australia are considered to be potentially related to enhanced fluid gradients and flow caused by paleotectonic activity^53^.

### Resolving the sand spike enigma: towards a proposed formation mechanism

Dewatering of sediment, e.g., during the passage of seismic waves or gravitational slumping, can initiate differential compaction within the sediment^17^ affected by the transient pressure pulse. The dewatered and, thereafter, compacted sediment bodies will eventually form an internally structureless^11,56^ and at least weakly solidified sand body, but will remain plastic for a period of time. This is in agreement with the observation that some sand spike individuals plastically deformed adjacent sand spikes^8^ (Fig. 5), which does not only demonstrate their (temporarily) ductile nature, but also a protracted, polyphase formation mechanism that produces the sand spikes. This dynamic formation mode is compatible with a scenario in which seismic waves (P- and S-waves followed by surface waves) travel across a large (basin-scale) sediment volume. Open pore space in the sediment, stripped off its fluids, is then readily cemented. The spikes are likely generated after the passage of the slowest seismic (Love and Rayleigh) waves, because the majority of spikes in the NAFB and at Mount Signal appear intact, preserving even the most delicate structural features^1–9^ (Fig. 5). Transitional forms of sand spikes resembling associated clastic sills and dikes also support their origin as a form of seismites^11,38^. At Untereichen, board- and ledge-like sand spike aggregates parallel to the host sediment layering are commonly vertically interconnected by dike-like features (Fig. 4a). Some isolated vertical dikes from the Hochgeländ also resemble sand spikes (Fig. 4d) and seem to represent a transitional type of sandstone body. These dike-like structures may represent “missing links” between sand spikes and clastic dikes and sills. Although the dominant process for the formation of sand spikes appears to be dewatering, the formation of dikes and sills requires the fluidization and subsequent injection of liquefied clastic material during the buildup of hydraulic overpressure^37^. We argue that sand spikes, which are globally rare compared to other types of sand injectites, represent the transitional form between classical dewatering structures (e.g., sand blows) and clastic injection dikes and sills^37–39^. The formation of sand blows requires a thick water-saturated sand body as parent material, whereas clastic injection dikes and sills can form from a less voluminous water-saturated body of sediment fluidized and injected into overlying dry(er) deposits^18^. From their mode of occurrence and structural setting, the formation of sand spikes seems to require loose and basically dry sands in which liquid water only occurs locally and/or along layer boundaries. This is in line with the observation that sand spikes commonly occur in board-like aggregates parallel to layer boundaries or in domains within seismites (e.g., folds) that were preferentially dewatered during the passage of a seismic wave. In the NAFB, sand spikes and classical dewatering structures are often found together in one outcrop where the spikes tend to show a preferred orientation along the pattern of other dewatering structures. Figures 3 and 4b, e, f show how sand spike specimens seem to preferentially cluster along folds within convolute bedding domains or slumps, where the local fluid concentration and fluid pressure may have been higher compared to the surrounding sediment.

The orientation of sand spike tails, which seems to be relatively uniform among individuals within one outcrop, was previously explained by their formation in context with the direction of the general (ground) water drainage system^2,6,8^. In our new interpretation of sand spikes as a form of seismite, spike tails turn out to be a reliable macroscopic paleoseismic indicator. In the proposed formation mechanism, the spike tails would grow in the direction of seismic wave propagation, i.e., away from the seismic source. This is convincingly demonstrated by the NAFB sand spikes stratigraphically and structurally linked to the Ries impact. Sand spike apices from locations south of the Ries crater are more or less uniformly orientated southward (allowing some natural variance), away from the center of the Ries crater (Fig. 1) and point to a direction almost perpendicular to the paleodrainage system^31,33–35^. In turn, regressing the orientation of radiating sand spike tails across the NAFB to a common area of origin at the Earth’s surface indicates the most likely seismic source, in this case the Ries crater region. This questions the formation of the NAFB sand spikes within the dynamic groundwater domain. In a similar manner, most sand spike tails at Mount Signal point west^2^, away from the Imperial/San Andreas Fault, a nearby major tectonic fault. Although a large number of major earthquakes must have affected southern California in the recent geologic past^57,58^, only the sand spike occurrence at Mount Signal (Fig. 6) has, thus far, been described in this region^2^. It can, therefore, be expected that the formation of sand spikes requires specific sediment and fluid properties within loose sands that are affected by seismic events.

### Sand, water, carbonate, and earthquakes: a dynamic recipe for sand spike formation

We propose sand spike formation is a complex, polyphase process associated with the formation of other (more typical) forms of seismites following seismically induced liquefaction and sediment dewatering^37–39^. As sand spikes occur preferentially along layer boundaries, they seem to form most readily in dry sands that still contain some water in layers or pockets. The compacted and subsequently dewatered sandy material of the sand spikes is initially ductile, but is stabilized and cemented by carbonate available within the surrounding sediment soon after spike formation. Liquefaction of water-saturated loose sands by earthquake-induced increasing pore-pressure usually leads to vertical or horizontal flow of the liquefied sediment and the rapid filling of temporary open fractures in the form of dikes and/or sills within the host sands^37^. The resulting dike or sill infill is usually dry and dense. Pore-pressure increase may be in parts responsible for the formation of sand spikes. However, short-distance transport of the liquefied sediment as far as ~1 m (i.e., the maximum length of most sand spikes) and the difference of the hydrostatic pressure between the liquefied sediment and the dry host deposits may not always be sufficient to produce typical clastic dikes. As demonstrated in the present study, transitional forms exist between sand spikes and sills or dikes (e.g., Figs. 3 and 4).

The eye-catching appearance and anatomy of sand spikes may, alternatively, suggest a genetic process completely different from the formation mechanism proposed for clastic dikes and sills. The typical shape of the sand spikes, with roundish and cauliflower-like heads and tail-like protrusions that point away from the seismic source, suggest water-filled pockets and layer boundaries in the host sands may have burst upon seismic compression. Recent work reveals that phase transitions of pore water may be an important process that operates during rapid (coseismic) slip induced by large earthquakes^59^. A mechanism referred to as coseismic boiling (a.k.a. decompression boiling or explosive flash vaporization) in earthquake-affected rocks^60,61^ may play an additional role. Fluids in the sediments affected by seismic waves can be decompressed to the point that water turns into vapor after the passage of those waves, leading to decompression-induced flash vaporization^60,61^. Cavity expansion along faults, joints, layer boundaries, and other lithologic inhomogeneities within the sediment can generate extreme reductions in pressure, facilitating localized formation of pore fluid vapor. After explosive decompression and flash vaporization, portions of the affected sediment are then absorbed into the transient cavities under temporary vacuum conditions^60^. Sand spike tails, however, suggest the sediment locally remains mobile, allowing portions of the liquefied sediment to flow in the direction of movement of the seismic waves in what may be referred to as an explosive steam jet during rapid expansion of water in the sediment. This complex but, presumably, short process (perhaps on the order of seconds?) could be responsible for strong compaction and a high degree of dewatering in the absorbed sediments that eventually wind up forming the conspicuous sand spikes that are still ductile before they are fully cemented. The solubility of minerals is strongly influenced by pressure and density of a fluid, and flash vaporization is thought to increase the degree of supersaturation by many orders of magnitude, resulting in near-instantaneous precipitation of cements upon pressure release^60^. The rapid initial precipitation of calcite, forming the sand spike cement, might stabilize the sand spikes shortly after their formation. That postseismic cementation process may also operate rapidly (perhaps within seconds to minutes?) and produce a skeletal cement framework, particularly within the porous spike tails. Secondary calcite cementation may last longer and further stabilize the sand spikes. Sediments underlying the Zapfensande in the NAFB, typically marls^4–6^, as well as surface-near water originating either from the karstified plateau of the emerging Swabian Alb to the North (with limestone-dominated alluvial fan deposits in the Mid-Miocene)^34^ or the Northern Calcareous Alps in the South^35^, may have been sources of carbonate within the Molasse basin. Free carbonate within the Zapfensande unit is, moreover, evidenced by the occurrence of authigenic calcite roses found, e.g., at Ochsenhausen^6^.

This being a preliminary explanation and novel working hypothesis for the formation of sand spikes—a long-standing geologic riddle—we also note that the aforementioned hypotheses and processes ought to be tested in the laboratory by wet-sand experiments and/or numerical simulations, using starting materials and parameters similar to those within the NAFB and at Mount Signal and a (strong) seismic point source at a certain distance from those water-saturated deposits. It is, in fact, possible that the non-lithified versions of sand spikes may have already been created in soft-sediment laboratories but remained unrecognized. On the other hand, basin-scale distances between the seismic source and the resultant sand spikes, as in the NAFB and the Imperial Valley of California, combined with a potentially strong seismic threshold magnitude required for sand spike formation, also beg the question whether sand spikes can be reproduced artificially in smaller-scale laboratory settings and with seismic shock sources of limited energy output. An additional approach towards the formation and growth of sand spikes in wet siliciclastic environments may be the dedicated analysis of select samples by means of carbon and oxygen isotopes (notably clumped isotope thermometry)^62^. However, assuming the formation of sand spikes is a rapid process (as opposed to the slow growth of carbonate concretions), as proposed in this paper, one would have to take into account that disequilibrium conditions between fluids and the carbonate cement, as well as the possible precipitation of primary (skeletal) vs. secondary carbonate, may affect isotope results and their interpretation. This type of investigation should, in any case, be performed following detailed sediment petrologic analysis on a (sub-)microscopic scale.

### Sand spikes as indicators for intense palaeoseismicity? The Ries impact and the San Andreas fault as major seismic triggers

In the first attempt by Rühl^5^ to link the Zapfensande in the NAFB with a potential seismic event, Alpine tectonics were the most promising triggering mechanism (the Ries crater, still thought to be of volcanic origin until 1961^63^, had not been considered to be a potential seismic source). A new structural-geologic look at the Zapfensande supports the novel notion that the sand spikes were formed as part of an at least ~10–15 m-thick Ries seismite unit, within a very narrow stratigraphic level^16^, in response to the earthquake triggered by the Ries impact. Likewise, sand spikes at Mount Signal seem to be genetically linked to the Imperial Fault as part of the San Andreas fault system^57,58^ that lies only some 40 km to the east^2^ (Fig. 6). This suggests those sand spikes, which almost uniformly point away from the Imperial fault, were generated during at least one episode of strong seismic shaking. This interpretation is in line with the earlier suggestions that sand spikes may be—one way or another—of tectonic origin^5,53^.

It is important to note that the timing of sand spike formation in the NAFB is not only stratigraphically consistent with the age of the Ries impact in the Mid-Miocene (Langhian)^20,21^, but also at odds with the timeline of active tectonism and seismicity associated with the Alpine orogeny^64^. Evidence for intense Alpine tectonic activity in the form of seismites occurs in Oligocene and Lower Miocene deposits within the NAFB. Dewatering structures occur near the Alpine front in the southern NAFB^15,16,65^. Major paleotectonic events are recorded in the uppermost Lower Marine Molasse (~24.8 Ma), the basal Lower Freshwater Molasse (~24.8 to 20 Ma), and in the Upper Marine Molasse (~19 to 17.5 Ma). No major seismic events are evident in the NAFB after 17.5 Ma^64^ and, accordingly, the UFM is generally unaffected by Alpine folding^33–35^. The Mid-Miocene decline in Alpine tectonic activity is coupled with decreasing subsidence in the NAFB, marked by the so-called “pre-Riesian hiatus” around 16 Ma^33^. A terminal Alpine seismic pulse was recognized in the Swiss Molasse, well before the Ries event^64^. In essence, by the time the Zapfensande were deposited, active Alpine thrusting and associated tectonism had already waned^19,33,64^.

Moreover, paleoseismicity within and around the Alpine orogen was quite distant from the seismite units found particularly in the Biberach/Hochgeländ area, which makes a genetic relationship between major Alpine earthquakes and soft-sediment deformation that far north in the NAFB during the Mid-Miocene implausible. Although seismites produced by Alpine tectonism are known in the older Molasse sediments, their geographic distribution is limited to an area at least ~50–100 km south of the seismite outcrops described in this study^15,16^. Likewise, tectonic activity associated with larger-scale tectonic graben and lineament structures within the Southern German crustal block (i.e., fault swarms in the Upper Rhine Graben, Hohenzollern-/Lauchert Graben, and Swabian Lineament directions) and intracontinental volcanic activity (e.g., the Neogene Kaiserstuhl, Hegau, and Urach-Kirchheim volcanic fields)^66,67^ were not energetic enough to cause widespread sediment deformation within the Molasse basin^15,16^.

In summary, the only seismic event strong enough to alter large volumes of sediment within the central and western Molasse Basin in the Mid-Miocene is the earthquake triggered by the Ries impact, with an estimated magnitude of *M*_W_ ~ 8.5^10,15,16^. The present study links the occurrence of sand spikes in the NAFB with the Ries event both structurally (spike tails point away from the Ries crater) and stratigraphically (the Zapfenande occur in a narrow Ries seismite interval^16^). The Ries seismite in the NAFB occurs at a stratigraphic level within the uppermost ~10–15 m of sediment at the time of impact, evidenced by distal Ries ejecta that blanket the seismite and mark the paleo-land surface^15,16,25^. All sand spike occurrences are situated in unconsolidated sediments that were deposited shortly before the impact event within a distance of up to ~110 km from the center of the Ries crater (Fig. 1). The age of the sand spike-hosting deposits near Biberach, Ulm, Günzburg, and Untereichen can be correlated with other Zapfensande sites in the NAFB by their fossil assemblage (European Land Mammal Zone transition MN 5 to MN 6^25,32,33^), i.e., the time immediately before the Ries impact (Fig. 2). The sand spike-bearing Zapfensande, unique within the Molasse basin, represent an important seismic event marker horizon within the NAFB and proves that it must have been produced by an extraordinary earthquake that surpassed the intensity of other endogenic tectonic events in this part of Central Europe.

We suggest sand spike formation requires a special combination of environmental conditions, including intense seismicity, specific properties of the affected sediments that, ideally, consist of unconsolidated sands, and host sediments that are only locally saturated with fluids. Sand spikes are, therefore, not expected to be a common and ubiquitous geologic feature. Likewise, sand spike-hosting deposits, due to their soft and friable nature, do not seem to possess a great potential for preservation over geologic time. Nevertheless, new sand spike occurrences may be discovered in the future (e.g., Supplementary Table 1) and sand spikes produced by the Mid-Miocene Ries impact-earthquake did survive with excellent exposures across the NAFB. Where sand spikes have survived erosional processes and occur in their original position, they represent a promising tool to identify strong earthquakes within tens to more than a 100 km palaeodistance and presumably within a relatively narrow stratigraphic interval. Not only do sand spikes in the sedimentary record indicate strong palaeoseismicity; they also seem to pinpoint the direction of the seismic source (compare Figs. 1 and 6).

The earthquake strength required to produce sand spikes is likely greater than magnitude *M*_W_ ~ 7. That is, sand spikes only seem to form during very energetic and destructive earthquakes. In the NAFB, sand spikes were formed in unconsolidated sands during the Ries impact-earthquake, which had an estimated magnitude of *M*_W_ ~ 8.5 or somewhat higher^10,15,16,19^. In contrast, no sand spikes have been recognized in association with the nearby Steinheim impact-earthquake that probably had a magnitude of about *M*_W_ 7^10,11^ and occurred in the same region ~0.5 Myr after the Ries impact^16^, which might indicate the lower threshold for sand spike formation. However, both climate and near-surface sediment conditions in the NAFB were significantly different during the two impacts^68,69^, which underlines how condition-sensitive the formation of sand spikes may be in nature.

Several strong historic earthquakes occurred along the San Andreas fault, which according to computer models can produce earthquakes of magnitudes *M*_W_ > 8^57,58^. As only one locality with sand spikes is known in the vicinity of the San Andreas fault, one can assume that only the strongest earthquakes may be capable of producing sand spikes. In turn, the identification of sand spikes near active fault systems can help reconstruct strong palaeoseismicity and allows to pinpoint the direction of the palaeoseismic source. This also helps assess the maximum seismic and related natural hazard potential of specific regions surrounding autochthonous sand spike occurrences at distances several tens to over a 100 km away from the sand spikes.

## Methods

### Field studies

In the last three decades (starting in 1993), natural outcrops, construction sites, and sand pits in UFM deposits in the western and central part of the NAFB deposits were systematically investigated on their occurrence of seismite horizons (in the form of dewatering structures and clastic dikes), and distal Ries ejecta horizons. In the last years, we expanded our investigations in these outcrops on the search for sand spike-hosting sand deposits. We paid particular attention to outcrops and ravines in the areas of Ravensburg, Biberach, Ulm, Günzburg, and Augsburg in SW Germany. After heavy rainfall in the last years in the Biberach and Ravensburg area, deposits with soft-sediment deformation structures, clastic dikes, and sand spikes were partially exposed below the distal ejecta horizon^15,16,25^ along ravine slopes. The structures were excavated during a large number of field campaigns in the years 2019 to 2021. We excavated sandy NAFB deposits over tens of meters vertically along the flanks of the “Tobel Oelhalde-Nord,” “Tobel Oelhalde-Süd,” “Josefstobel,” and “Kleintobel,” and others over several tens of meters laterally along the flanks of the ravines. Additional field investigations were carried out in sand pits (e.g., Thierhaupten, Untereichen) over the last two decades. Sand spike orientation (azimuth/bearing of spike tail) data were acquired using a geological compass at Ochsenhausen, in the Hochgeländ area, at Untereichen, and at Thierhaupten (see Fig. 1 and Supplementary file). Historic finds of sand spikes in the NAFB^6^ sampled in private collections and in the local Braith-Mali-Musuem in Biberach a. d. Riss were also analyzed.

### Petrography

Sand spike specimens in particular from Ochsenhausen were cut, stabilized by synthetic resin, and processed to polished thin sections for supporting optical and electron beam analysis. Standard petrographic analyses were carried out using an optical polarization microscope and a CamScan SC44 scanning electron microscope coupled to a semiquantitative EDAX PV 9723/10 energy-dispersive X-ray system at the Universität of Stuttgart (operating conditions 15–20 kV accelerating voltage).

## Supplementary information

Supplementary Information

## Data Availability

All data generated or analyzed during this study are included in this published article and its Supplementary Information file.
