# Supplementary material for: Sand spikes pinpoint powerful palaeoseismicity
Source: Nat Commun. 2021 Nov 18;12:6731. doi: 10.1038/s41467-021-27061-6 (PMC8602635; doi:10.1038/s41467-021-27061-6)
Supplement: Supplementary file 1 — Supplementary Information [file 41467_2021_27061_MOESM1_ESM.pdf]

Supplementary Material to:

Sand spikes pinpoint powerful palaeoseismicity

Elmar Buchner*[1,2], Volker J. Sach[2,3] & Martin Schmieder[1,2]

[1]HNU - Neu-Ulm University of Applied Sciences, Wileystraße 1, D-89231 Neu-Ulm, Germany; E-mail: elmar.buchner@hnu.de

[2]Meteorkrater-Museum Steinheim, D-89555 Steinheim am Albuch, Germany

[3]Fokus Natur, Am Heselsberg 29, D-88416 Ochsenhausen, Germany;

E-Mail: vsach@gmx.de

E-Mail: martin.schmieder@hnu.de

THIS FILE INCLUDES:

1. Supplementary Note 1
2. Supplementary Figures 1 to 5
3. Supplementary Table 1
4. Supplementary References

SUPPLEMENTARY NOTE 1

Description of outcrops

Ravine, ‘Tobel Oelhalde-Süd‘, Hochgeländ south of Biberach an der Riß;

Coordinates: 48º02’16.08’’N, 09º49’47.06’’E

Several (temporary) natural outcrops along ravines occur in the Hochgeländ south of Biberach (Fig. 1), one of which is the Ravine ‘Tobel Oelhalde-Süd’. The deposits of the Fluviatile Untere Serie as part of the Upper Freshwater Molasse[1-8] that is dominated by fine sands, crop out at around 594-608 m a.s.l. At the northern flank of the ravine, two horizons of distal Ries ejecta crop out at a height of ~605.3 and 606.7 m a.s.l., respectively. The upper (reworked) horizon exclusively consists of small fragments of Upper Jurassic rocks. Larger limestone components, some of which are shatter-coned, characterize the primary, non-reworked, lower horizon of distal Ries ejecta at 605.3 m a.s.l.[1,3-6] (see detailed description therein). All seismite- and sand spike-hosting deposits are stratigraphically located below the distal Ries ejecta horizon and were, thus, deposited shortly before the Ries impact occurred.
A seismite horizon of about 10 m thickness that directly underlies distal Ries ejecta, exhibits various distinct dewatering structures. In the lower parts of this horizon, convolute bedding, flame structures, and ball-and-pillow structures occur. Large slump structures dominate the upper parts of the seismite horizon. Distinct sand spikes also appear within the seismite horizon in a height of 595-605 m a.s.l. as board-like aggregates formed parallel to the layer boundaries or as single arrow-like individuals. The sand spikes obviously follow the pre-existing lineation of the dewatering structures and are particularly concentrated in faults of convolute bedding (Fig. 3 d,e; Fig. 4 e,f; Supplementary Fig. 1). Nearly all sand spikes in this ravine are orientated NNW to SSE (with a variance NW/NNW to SE/SSE). The apices of the sand spikes point towards SSW. Some sand spikes also occur in the adjacent ravines within seismite horizons (e.g., Tobel Oelhald-Nord[9,10], but their orientation is usually also NNW to SSE with a variance of NW/NNW to SE/SSE). The orientation of sand spikes in the Hochgeländ area is generally N to S but slightly varies from outcrop to outcrop (Fig. 1).

Former ‘Liebherr outcrop’ near Ochsenhausen, approximately 10 km east of ‘Hochgeländ’;

Coordinates: 48°04′05.5″N, 9°58′10.6″E

In the temporary construction site ‘Liebherr outcrop’ near Ochsenhausen, a suite of at least 2.5 m thickness of sandy sediments of Upper Freshwater Molasse deposits were temporarily exposed around the year 1995[9,11]. Flame structures and intensely faulted slumps were discovered in these deposits at around 600 m a.s.l.[9]. Fold axes of the metre-scale slumps strike in a WNW-ESE direction[12] (see detailed description of the seismites of this outcrop therein). The Ochsenhausen sediments stratigraphically belong to the Untere Fluviatile Serie[1,2,7,8]. According to the results presented in two recent studies by our group[9,10,13], soft-sediment deformation in the Upper Freshwater Molasse deposits can be associated with a seismic event triggered by the Ries impact.

An outcrop about 10 m wide and 4-5 m in height showing large horizontally moved slumps (Supplementary Fig. 2) was already recorded by one of the authors (V.J.S) in the year 1993 in an area which now hosts the Liebherr factory in Ochsenhausen but is firstly presented in the frame of the present study. The slumps occur in the same topographic level as the sand deposits with slumps and convolute bedding mentioned in an earlier study[9] as well as in the same level as the 5 m thick sand spike-hosting deposits shown in Fig. 2c. The sand spikes of this outcrop occur as board-like aggregates or as arrow-like individuals and are explained in detail in the main text of the study as well as in Fig. 6 and Supplementary Fig. 4 c-e). These features from Ochsenhausen often exhibit exemplary forms for sand spikes and are stored at the Local Museum in Biberach an der Riß (‘Braith-Mali-Museum’) and in many private collections (Supplementary Fig. 4 c-e).

Sand and clay pit Untereichen-Altenstadt south of Ulm;

Coordinates: 48°10′25.5″N, 10°06′50.6″E

The sedimentary succession of Altenstadt-Untereichen (Fig. 3 a,b; Fig. 4 a-c; Supplementary Fig. 3) comprises of a 30-m thick marly (Limnische Untere Serie) and an up to 15-m thick sandy unit (Fluviatile Untere Serie[1,2,7,8]) of the Upper Freshwater Molasse deposits in the central North Alpine Foreland Basin. The latter deposits occur around 560 to 575 m a.s.l.. The sandy sequence is largely composed of medium to fine-grained sand and only a few thin laminated clay deposits of abandoned channels have been detected[7,8]. The sandy sediments are built up by braided river deposits[7,8]. Sand spikes are very abundant in the entire 15 m thick sequence. They often occur as board-like aggregates formed parallel to the boundary layers or as arrow- or nail-like individuals. Generally, the sand spikes occur in apparently undisturbed deposits, however, convolute bedding can be observed in parts of the sediments, in particular at the northern part of the recent sand pit Untereichen. Water

escape structures were also reported in the literature[8] but interpreted as the result of high sedimentation rates in the fluvial channels. Many thin clastic dikes cut through the sandy deposits of the Fluviatile Untere Serie (Supplementary Fig. 3).

SUPPLEMENTARY FIGURE 1

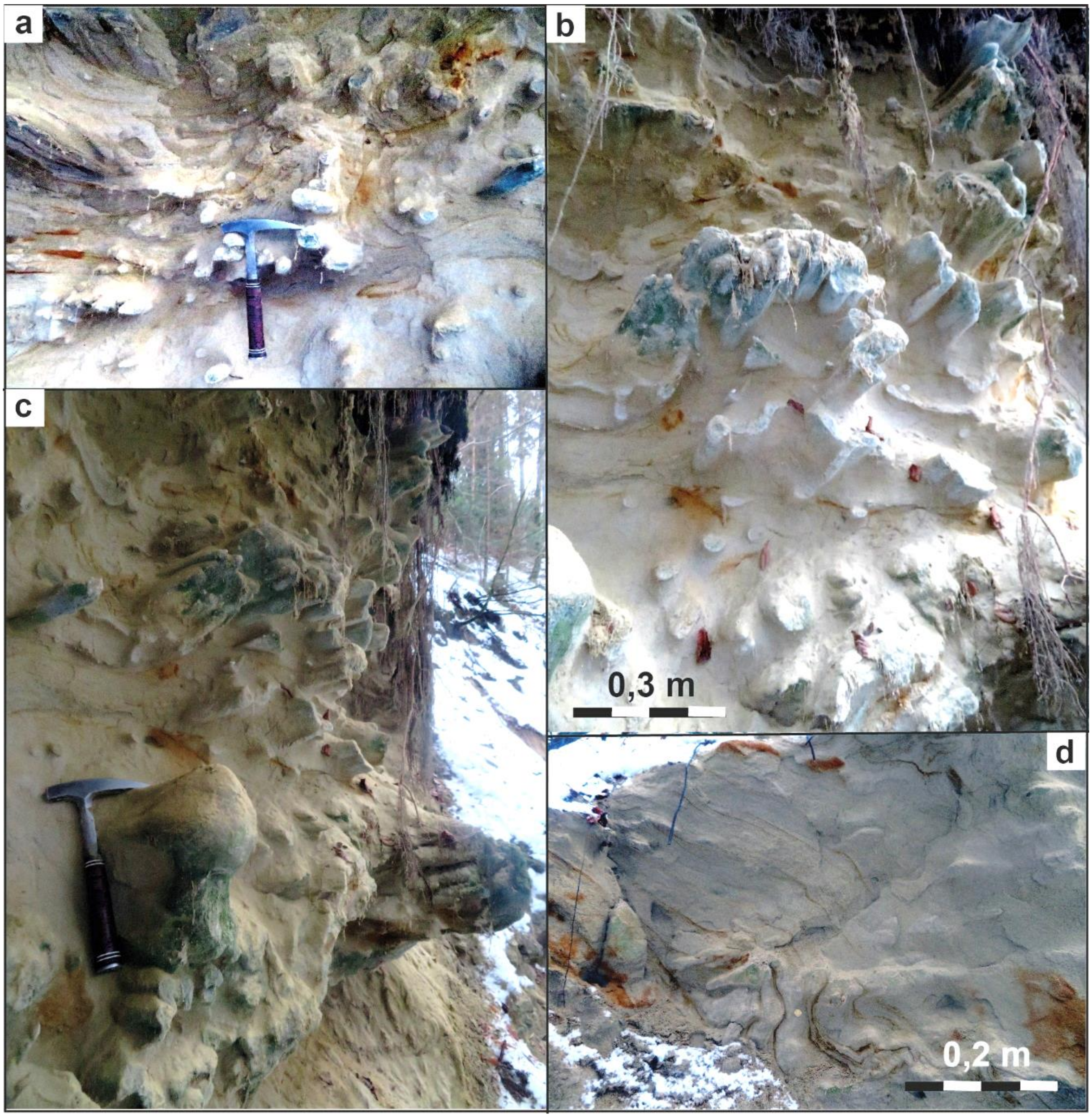

Supplementary Figure 1: Tobel Oelhalde-Süd, Hochgeländ near Biberach: a and b: Sand spikes as single individuals and aggregates in Upper Freshwater Molasse deposits (Fluviatile Untere Serie) showing convolute bedding. Note that sand spike occurrence is obviously orientated along the convolute bedding structures. c: Sand spikes as single individuals and aggregates in sandy deposits that exhibit convolute bedding, small slumps, and sand diapirs. d: Convolute bedding in sandy deposits free of sand spikes in the same outcrop (all photographs taken by V.J.S. in January 2021).

SUPPLEMENTARY FIGURE 2

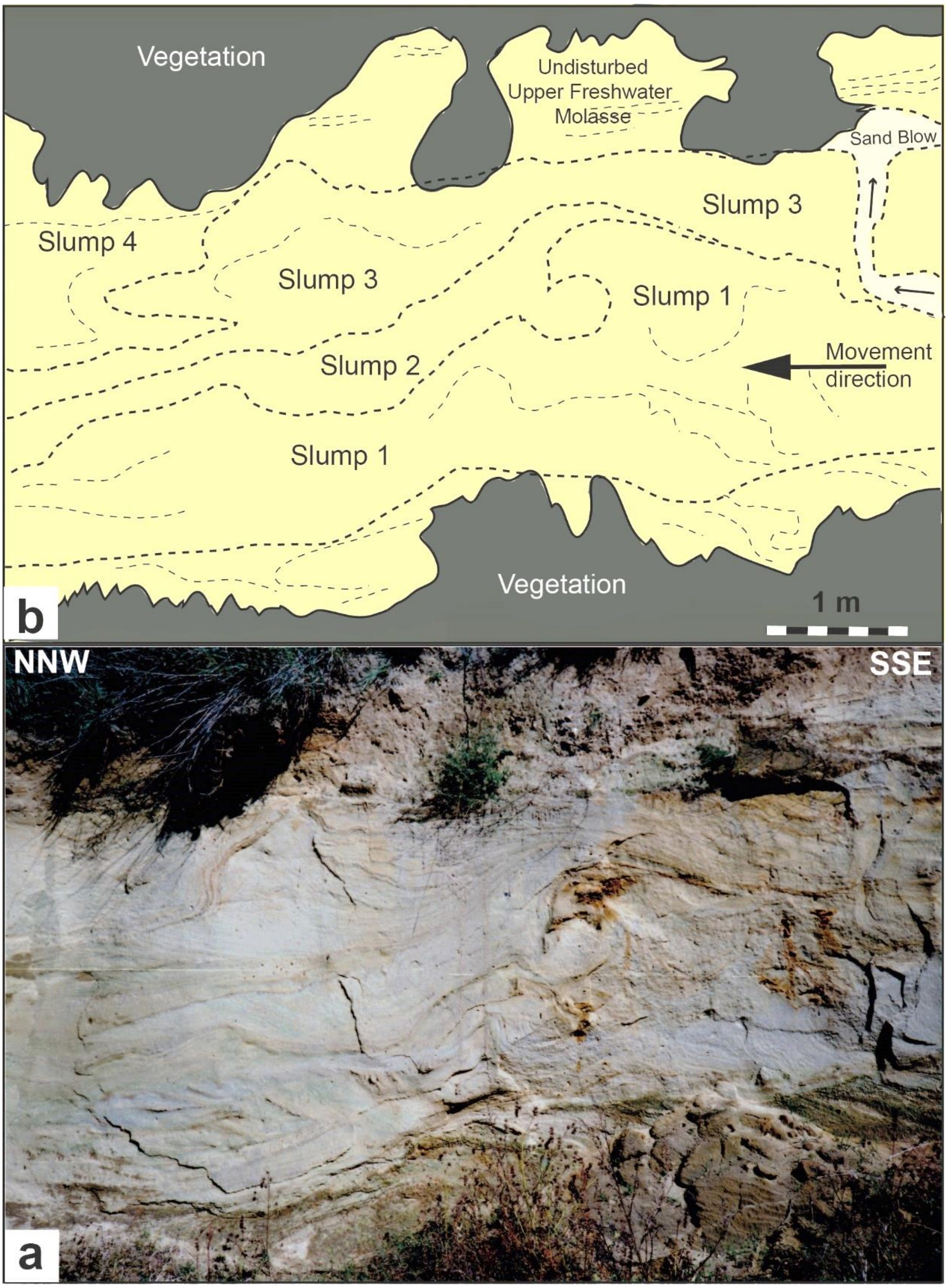

Supplementary Figure 2: Temporary outcrop (a) at Liebherr construction site (situation in 1993) in sandy deposits of the Upper Freshwater Molasse (Fluviatile Untere Serie) showing a Ries seismite horizon with at least four slump structures (b). The outcrop is situated in the same stratigraphic and topographic level as the Ries seismite horizon described in two recent studies[9,10] and as the Ries sand

spike-hosting deposits cropping out some tens of metres away (photograph taken by V.J.S. in summer of 1993).

SUPPLEMENTARY FIGURE 3

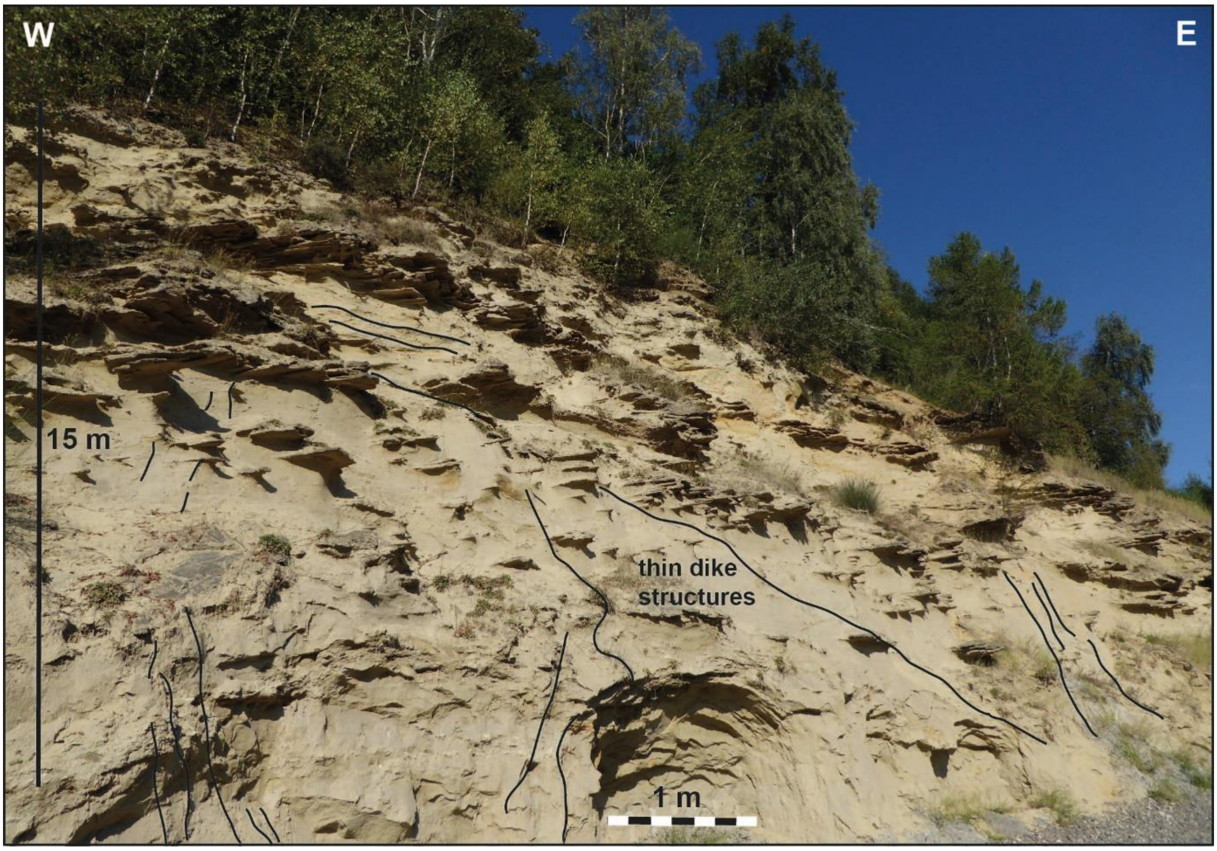

Supplementary Figure 3: Sand and clay pit Altenstadt-Untereichen south of Ulm. The Upper Freshwater Molasse deposits (Fluviatile Untere Serie) exhibit an about 15 m thick horizon of sand-spike-hosting sands; sand spikes appears board-like aggregates formed parallel to the layer boundaries and single arrow-like individuals. The sandy deposits partially show convolute bedding, dewatering structures, and small clastic dikes (photograph taken by V.J.S. in summer of 2016).

SUPPLEMENTARY FIGURE 4

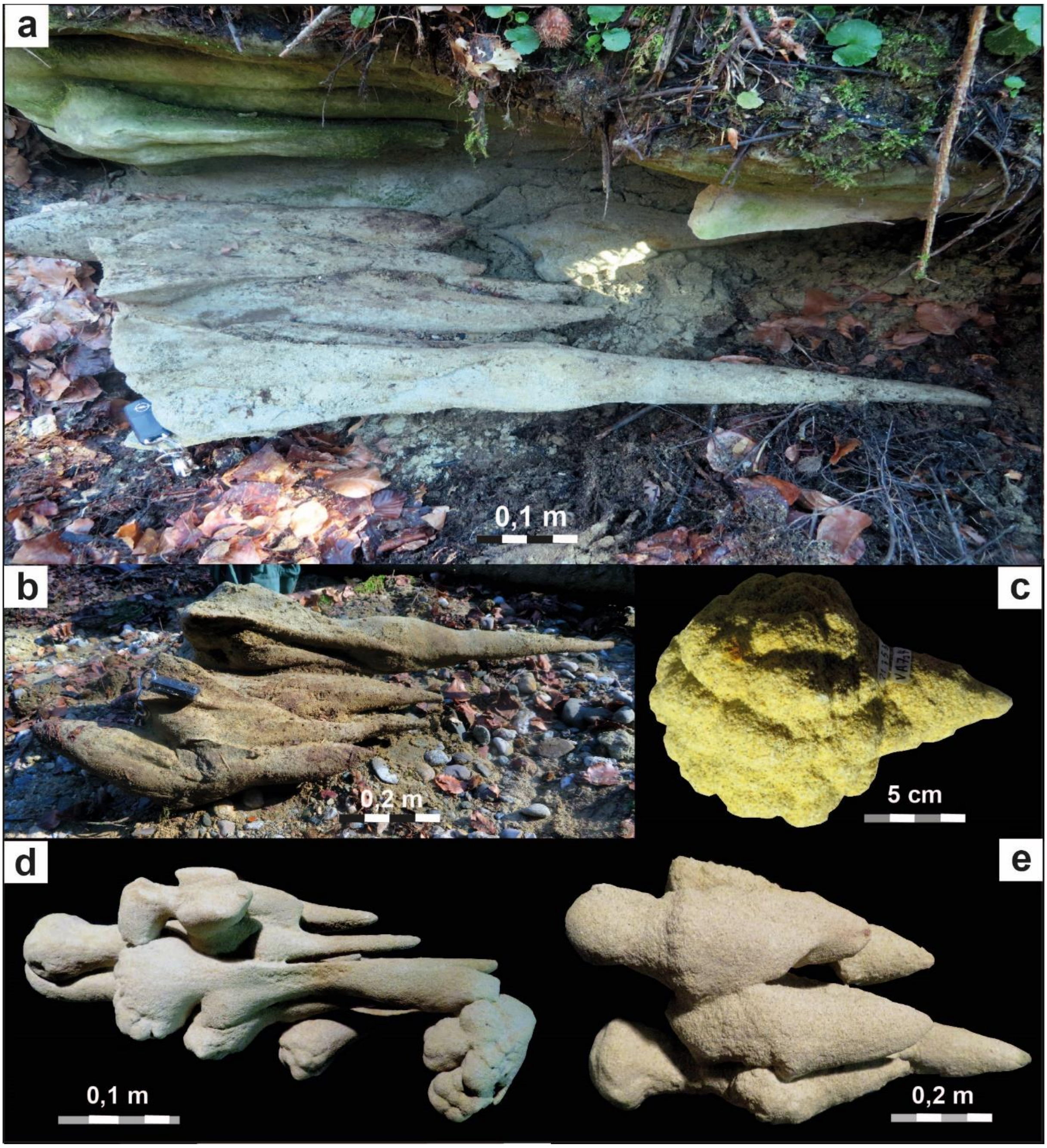

Supplementary Fig. 4: Variance of outer appearance of sand spikes. a and b: Sand spikes from Josefstobel, Hochgeländ near Biberach. These aggregates of sand spikes show a more tube-like form. c: A short chubby sand spike individual showing a cauliflower-like head and an extremely short stick. d: Sand spike aggregate with more flat heads. e: Sand spike individuals that spear other individuals forming a conspicuous mushroom-like aggregate. c to e: Sand spikes sampled at former ‘Liebherr construction site’ Ochsenhausen and stored in the Local Museum at Biberach an der Riß (‘Braith-Mali-Museum’) (all photographs taken by V.J.S. in springtime of 2021).

SUPPLEMENTARY FIGURE 5

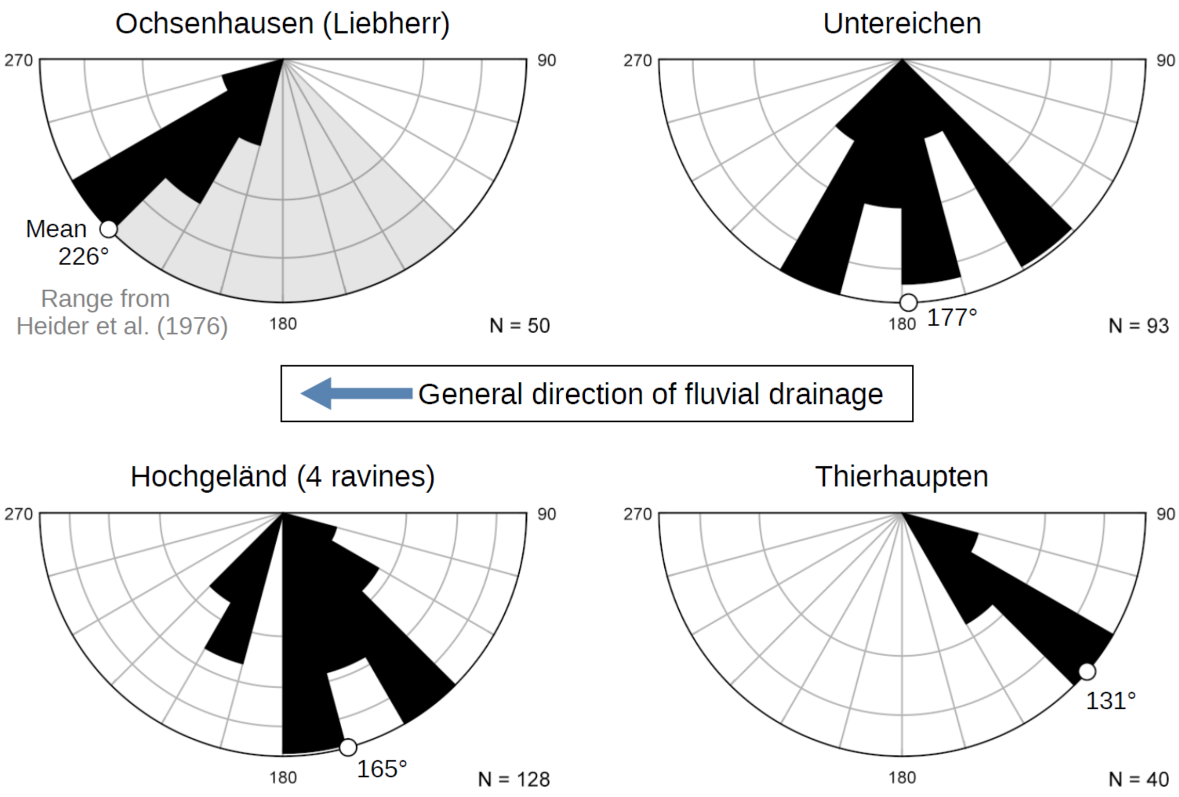

Supplementary Fig. 5: Orientation (azimuth) of sand spike tails (black segments) at four major sand spike occurrences within the North Alpine Foreland Basin. Data for the former Liebherr outcrop at Ochsenhausen (V.J.S., 1993), some of those reported for Untereichen, and those for Thierhaupten (E.B., 2001/2002) are legacy data. Data for the Hochgeländ were obtained recently (V.J.S., 2021). The gray shaded range shown for Ochsenhausen represents the orientation of sand spike tails reported in an older study[11]. Small circles indicate the mean spike tail azimuth for each sand spike site. The main direction of Mid-Miocene fluvial drainage was from the east to the west[14,15,16] (blue arrow).

SUPPLEMENTARY TABLE 1

Table 1: Summary of additional localities with deposits hosting sand spikes and sand spike-like sedimentary features.

**Further localities with potential sand spikes**	**Appearance**	**Age**	**References (No. of reference in the reference list of the main text in brackets**
Fontainebleau (SE of Paris), France	Sand spike-like features; single and combined specimen	Oligocene and Holocene unconsolidated sands	(17) Plaziat, J.C. & Poisson, M.A. (1992) Mise en évidence de plusieurs séismes majeurs dans le Stampien supérieur continental au sud de Paris: enregistrements sédimentaires de la tectonique Oligocène. Bulletin de la Société Géologique de France 163 (5): 541–551.
Saint-Fons near Lyon, Département du Rhône, France	Sand spike-like features; single and layered specimen	Miocene unconsolidated sands	(18) Thomas, P. (2020) Les flute casts des “molasses” miocènes de la région lyonnaise (Rhône) (https://planet-terre.ens-lyon.fr/image-de-la-semaine/Img691-2020-11-09.xml)
Basilicata coast of the Ionian Sea, south Italy	Sand spike-like features; single specimen	Pleistocene unconsolidated sands	(19) McBride, E.F., Picard, M.D., & Folk, R.L. (1994). Orientated Concretions, Ionian Coast, Italy: Evidence of Groundwater flow direction. Journal of Sedimentary Research, A64, 535-540.
Crotone basin, south Italy	Sand spike-like features; sand spike packages	Middle Pliocene to Pleistocene unconsolidated sands	(20) Balsamo, F., Storti, F. & D. R. Gröcke, D.R. (2013) Fault-related fluid flow history in shallow marine sediments from carbonate concretions, Crotone basin, south Italy. Journal of the Geological Society, London, Vol. 169, 2012, pp. 613 –626. doi: 10.1144/0016-76492011-109.
South-Central Utah, U.S.A. (Navajo Sandstone)	Sand spike-like features; single and combined specimen	Jurassic consolidated sandstone	(21) Loope, D.B., Kettler, R.M.& Weber, K.A. (2011) Morphologic Clues to the Origins of Iron Oxide–Cemented Spheroids, Boxworks, and Pipelike Concretions, Navajo Sandstone of South-Central Utah, U.S.A." Faculty Publications in the Biological Sciences, 198, University of Nebraska.
Western Nebraska and eastern Wyoming, USA (Arikaree)	So-called ‘pipy concretions’ of the Arikaree	Miocene Marsland Formation	(22) Schultz, C. B. (1941) The Pipy Concretions of the Arikaree. Bulletin of the University of Nebraska State Museum 23, 15 p. (his Fig. 36, lower left)
Central New Mexico, USA (Sierra Ladrones Formation)	Sand spike-like features; single and combined specimen	Pliocene-Pleistocene unconsolidated sands	(23) Davis, J.M. (1999) Oriented carbonate concretions in a paleoaquifer: Insights into geologic controls on fluid flow. Water Resources Research, 35, 1705-1711.
Cape Liptrap, Victoria, Australia	Sand spike-like features; single and combined specimen	Pleistocene unconsolidated sands	(24) McCullough, L.N. (2003). Habit, Formation, and implications of elongated, calcite concretions, Victoria, Australia. Department of Geology Wittenberg University, Ohio, USA. Senior Honors Thesis.

SUPPLEMENTARY REFERENCES

1. Sach, V. J. Neue Vorkommen von Brockhorizonten in der Oberen Süßwassermolasse von Baden-Württemberg (Deutschland) – Zeugnisse der Rieskatastrophe im Mittelmiozän. *N. Jb. Geol. Paläont. Abh. 205*, 323-337 (1997).
2. Sach, V. J. Litho- und biostratigraphische Untersuchungen in der Oberen Süßwassermolasse des Landkreises Biberach an der Riß (Oberschwaben). *Stuttg. Beitr. Naturkde.* **B 276**, 167 pp. (1999).
3. Buchner, E. et al. Simulation of trajectories and maximum reach of distal impact ejecta under terrestrial conditions: consequences for the Ries crater, southern Germany. *Icarus* **191**, 360–370 (2007).
4. Sach, V. J. Strahlenkalke (Shatter-Cones) aus dem Brockhorizont der Oberen Süßwassermolasse in Oberschwaben (Südwestdeutschland) – Fernauswürflinge des Nördlinger-Ries-Impaktes (Pfeil, Munich, 2014).
5. Letsch, D. Diamictites and soft sediment deformation related to the Ries (ca. 14.9 Ma) meteorite impact: the "Blockhorizont" of Bernhardzell (Eastern Switzerland). *Intern. J. Earth Sci*. **107**, 1379–1380 (2017).
6. Holm-Alwmark, S., Alwmark, C., Ferrière, L., Meier, M.M.M., Lindström, S. Kenny, G.G., Sheldon, E. , Schweigert, G., Spötl, C. , Whitehouse, C. & Hofmann, B. Shocked quartz in distal ejecta from the Ries impact event (Germany) found at ~ 180 km distance, near Bernhardzell, eastern Switzerland. *Nature Sci. Rep.* **11**:7438 (2021).
7. Maurer, H. & Buchner. E. Rekonstruktion fluvialer Systeme der Oberen Süßwassermolasse im Nordalpinen Vorlandbecken SW-Deutschlands. *Z. dt. Ges. Geowiss.* **158**, 249-270 (2007).
8. Prieto, J., Böhme, M., Maurer, H., Heissig, K. & Abdul Aziz, H. Biostratigraphy and sedimentology of the Fluviatile Untere Serie (Early and Middle Miocene) in the central part of the North Alpine Foreland Basin: implications for palaeoenvironment and climate. Intern. *J. Earth Sci.* **98**, 1767–1791 (2009).
9. Sach, V. J., Buchner, E. & Schmieder, M. Enigmatic earthquake-generated large-scale clastic dyke in the Biberach area (SW Germany). *Sed. Geol.* **398**, 105571 (2020).
10. Buchner, E., Sach, V. J. & Schmieder, M. New discovery of two seismite horizons challenges the Ries–Steinheim double-impact theory. *Nature Sci. Rep.* **10**:22143 (2020).
11. Heider, J., Wegele, A. & Amstutz, G. C. Beobachtungen über Sandrosen und Zapfensande aus der Süßwassermolasse Südwürttembergers. *Aufschluß* **27**, 297-307 (1976).
12. Tohver, E. et al. End-Permian impactogenic earthquake and tsunami deposits in the intracratonic Paraná Basin of Brazil. *GSA Bull.* **130**, 1099–1120 (2018).

13. Schmieder, M., Sach, V.J. and Buchner, E., The Chöpfi pinnacles near Winterthur, Switzerland: long-distance effects of the Ries impact-earthquake? *Int. J. Earth Sci.,* 3 p., doi: 10.1007/s00531-021-02082-0 (2021).
14. Geyer, O. F. & Gwinner, M. P., Geologie von Baden-Württemberg. Schweizerbart, Stuttgart, 5th ed., 627 p. (2011).
15. Doppler, H. J., Heissig, K. & Reichenbacher, B. Die Gliederung des Tertiärs im süddeutschen Molassebecken. *Newsl. Stratigr.* **41**, 359–375 (2005).
16. Meschede, M. & Warr, L. N., The Geology of Germany – A Process-Oriented Approach. Springer, 304 p. (2019).
17. Plaziat, J.C. & Poisson, M.A. Mise en evidence de plusieurs seismes majeurs dans le Stampien superieur continental au sud de Paris; enregistrements sedimentaires de la tectonique Oligocene. *Bull. Soc. Géol. France* **163**, 541–551 (1992).
18. Thomas, P. Les flute casts des “molasses” miocènes de la région lyonnaise (Rhône) (https://planet-terre.ens-lyon.fr/image-de-la-semaine/Img691-2020-11-09.xml) (2020).
19. McBride, E.F., Picard, M.D., & Folk, R.L. Orientated Concretions, Ionian Coast, Italy: Evidence of Groundwater flow direction. *Journ. Sedim. Res*., **A64**, 535-540 (1994).
20. Balsamo, F., Storti, F. & Gröcke, D. R. Fault-related fluid flow history in shallow marine sediments from carbonate concretions, Crotone basin, south Italy. *J. Geol. Soc*., **169**, 613 –626 (2013).
21. Loope, D.B., Kettler, R.M. & Weber, K.A. Morphologic Clues to the Origins of Iron Oxide–Cemented Spheroids, Boxworks, and Pipelike Concretions, Navajo Sandstone of South-Central Utah, U.S.A. *Fac. Publ. Biol. Sci.,* **198**, 505-520 (2011).
22. Schultz, C. B., The Pipy Concretions of the Arikaree. Bull. Univ. Nebraska State Museum 2, 69-82 (1941).
23. Davis, J.M. Oriented carbonate concretions in a paleoaquifer: Insights into geologic controls on fluid flow. *Wat. Resourc. Res.* **35**, 1705-1711 (1999).
24. McCullough, L. N., Habit, formation, and implications of elongeate, calcite concretions, Victoria, Australia. Department of Geology, Wittenberg University, Ohio, USA. Published Senior Honors Thesis (2003).
